# An enhanced view on the Mediterranean Sea crust from potential fields data

**DOI:** 10.1038/s41598-023-35282-6

**Published:** 2023-05-23

**Authors:** Daniele Sampietro, Martina Capponi, Erwan Thébault, Lydie Gailler

**Affiliations:** 1 Geomatics Research & Development srl, 22074 Lomazzo, CO Italy; 2 Laboratoire Magmas et Volcans, CNRS, IRD, OPGC, Université Clermont Auvergne, 63170 Aubière, France

**Keywords:** Solid Earth sciences, Geophysics, Tectonics

## Abstract

The Earth’s crust is exceptionally important to understand the geological evolution of our planet and to access natural resources as minerals, critical raw materials, geothermal energy, water, hydrocarbons, etc.. However, in many regions of the world it is still poorly modelled and understood. Here we present the latest advance on three-dimensional modelling of the Mediterranean Sea crust based on freely available global gravity and magnetic field models. The proposed model, based on the inversion of gravity and magnetic field anomalies constrained by available a-priori information (such as interpreted seismic profiles, previous studies, etc.), provides, with an unprecedented spatial resolution of 15 km, the depths of the main modelled geological horizons (Plio-Quaternary, Messinian and Pre-Messinian sediments, crystalline crust and upper mantle), coherent with the known available constraints, together with the three-dimensional distribution of density and magnetic susceptibility. The inversion is carried out by means of a Bayesian algorithm, which allows to modify at the same time the geometries and the three dimensional distributions of density and magnetic susceptibility, always respecting the constraints introduced by the initial information. In addition to unveil the structure of the crust beneath the Mediterranean Sea, the present study also shows the informative content of freely available global gravity and magnetic models, thus putting the base for the development of future high resolution models of the Earth crust at global level.

## Introduction

The Mediterranean Sea region is probably one of the most studied areas worldwide. Its crust is particularly important for scientific reasons being the tectonic boundary between the African, the Eurasian and the Arabian plates, as well as for geohazard and economic reasons (mainly related to hydrocarbon exploration). Several recent studies on the Mediterranean Sea crust at national and local scales can be found in literature, e.g.^1–5^ but only few works cover the whole Mediterranean Sea region and usually date back to the end of the twentieth century. We recall here the work done in the framework of the International Bathymetric Chart of the Mediterranean intergovernmental project, by the Intergovernmental Oceanographic Commission of UNESCO (starting from 1972), that produced among other products, in more than 20 years of activities, maps of the Plio-Quaternary sediments thickness, of the seismicity and of recent sedimentation in the Mediterranean region^6^. Beside these studies, quantitative information on the structure of the Mediterranean Sea crust is available only through continental^7^ or global models^8–10^. These models generally show some drawbacks: either they are compilations of previous studies, with input data non homogeneously distributed in space and time^7^, or they are too coarse in terms of spatial resolution to provide detailed information^8–10^.

In our work we studied the whole Mediterranean Sea crust from $6^\circ 12^{\prime }$ West to $36^\circ 37^{\prime }$ East and from $29^\circ 45^{\prime }$ North to $46^\circ 5^{\prime }$ North, from the sea level to a depth of 50 km. Particular attention has been paid in the modelling of the crust in the offshore regions (which is the main objective of the current research), while the onshore area is reported here mainly for the sake of completeness.

Our result on the Mediterranean Sea crust is obtained from a joint Bayesian inversion of gravity and magnetic data, in which previous studies and seismic derived information, together with their expected accuracy, are used to constrain in a probabilistic way, the solution. The idea behind the used joint Bayesian inversion algorithm consists in computing from the available information, e.g. interpreted seismic profiles, previous studies, etc., a complete three-dimensional model of the volume (in the following called a-priori model) and in perturbing this model, according to the expected accuracy of each dataset used to build the model itself, trying to fit both gravity and magnetic potential fields. This will allow, contrary to many classical gravity or magnetic inversion schemes (such as^11,12^ and the many contributions derived from these two works), to estimate at the same time the geometries of the main geological units and their density and magnetic susceptibility distributions, keeping sharp realistic boundaries between different layers. The result, is a complete three dimensional model of the studied region, in terms of geological units, density and susceptibility distributions which is coherent with all the exploited a-priori information (i.e. it falls within the confidence intervals of the a-priori model) and also fits the observed gravity and magnetic data. We will call it a-posteriori model in the following.

We use the XGM2019e model^13^ and the WDMAM v2 model^14^ to derive gravity and magnetic fields respectively, synthesising both the models at an altitude of 4000 m. Note that, being both gravity and magnetic data available on a global scale, the inversion can be replicated in principle everywhere, thus allowing to estimate a high-resolution, high-accuracy crustal model of the whole Earth. In the following we present and discuss the data used to build the a-priori model and the main results of the performed inversion. The inversion algorithm is outlined in the Supplementary Information. The interested reader can find more details on the applied Bayesian inversion in^15–17^.

## Data

As summarised in the Introduction we derived the gravity observations from the XGM2019e model which in turns is computed combining high resolution (about 2 km) gravity from satellite radar altimetry offshore with a coarser dataset (spatial resolution of about 27 km) based on ground data onshore. The model is then completed onshore with forward-modelled topographic gravity anomalies^13^. An accuracy of 3 mGal, in terms of standard deviation (STD), with a spatial correlation length of 15 km (offshore) is assumed for the gravity grid accordingly to^18^. Considering the way in which the XGM2019e model is computed a lower spatial resolution is expected onshore. We synthesised the model on a grid at an altitude of 4 km (outside the masses) and removed from the initial dataset the effect of topography and bathymetry from^19^. Being interested in the Earth crust up to 50 km we removed from the reduced gravity field the gravitational effect of the density distribution between 50 km (maximum depth of the inverted model) and 300 km taken from^10^. The 300 km depth for this further reduction and its accuracy have been assessed in^18,20^.

For the magnetic field we used the WDMAM v2 model^14^ at an altitude of 4000 m. It is worth noting that the WDMAM v2 does not include geophysical a-priori information over the Mediterranean Sea crust^21^. The accuracy of the model on the Mediterranean Sea area has been assessed in^18^, so in the inversion we supposed an observation error of 23 nT (STD).

Given the accuracy of the gravity and magnetic observations we exploited the methodology proposed in^18^ to set the optimal size of the 3D volume: a discretization of the horizontal plane of 10 arcmin roughly corresponding to 15 km, was selected for the inversion for a total of about $260 \times 100$ cells. Regarding the vertical direction we selected a spatial resolution ($\Delta z$) ranging from 200 m at the top of the model (sea level) to 1200 m at the base of the model (50 km). The total number of cells is about $2\cdot 10^6$, corresponding to about $6\cdot 10^6$ unknowns (i.e. a label, a density and a magnetic susceptibility for each volumetric element).

In order to apply the Bayesian inversion the a-priori model should be created. To this aim we retrieved from literature the depth of main horizons, separating the different geological units in the studied volume, together with the expected density/magnetic susceptibility distributions of each unit and all the corresponding uncertainties. During the inversion, the accuracy will be used in order to allow possible variations of the initial geological horizons and density/susceptibility values. In detail, the considered horizons are the bathymetry, the base of Plio-Quaternary sediments, the base of Messinian sediments, the depth to basement (i.e. the boundary between the sedimentary layers and the crystalline crust) and the Moho. The model is completed by the Curie Point depth, i.e. the theoretical surface with a temperature of approximately 580 $^\circ$C representing the base of lithospheric magnetic sources. Starting from the bathymetry, it has been taken from^22^. To simplify the modelling, and being this layer nowadays known with a high level of accuracy, its geometries have been considered exact and fixed within the inversion process. The second layer has been obtained by adding to the bathymetry depth the thickness of Plio-Quaternary sediments derived from^23^, which in turn has been obtained by merging a digitised high resolution version of the IBCM-PQ map^6^ with interpreted seismic profiles and public data from^24–29^. Within^23^ also a formal accuracy, ranging between 100 m and 200 m (STD), is provided.

The base of Messinian sediments is obtained by integrating the work of^30^ with the seismic profiles described in^5,24,31–34^ by means of a kriging interpolation^35^. We started by geo-referencing and digitising the map in^30^ and the profiles provided in the above publications. The result of this operations is a set of sediment thicknesses distributed all over the study area. Starting from this dataset, we computed the empirical variogram and fitted it with a Stable variogram function^35^. We then gridded the dataset by means of ordinary kriging^35^. The depth of the base of this layer has been estimated by summing the obtained thickness to the depth of the base of the Plio-Quaternary sediments. An accuracy of about 300 m (STD) has been predicted by the kriging interpolation.

The following surface is the depth to basement, i.e. the boundary between the Pre-Messinian sediments and the crystalline crust. In the current work, the different Pre-Messinian sedimentary units have been modelled by means of a unique layer. This choice is justified first of all by the lack of knowledge on the sedimentary pack in several areas of the study region. Seismic acquisitions (both 2D and 3D) for resource explorations usually in fact do not reach the depth required to accurately model this sedimentary layer, as a consequence only few deep seismic profiles are available. Moreover, the different Pre-Messinian sedimentary layers can have very similar densities, thus making the possibility to define a clear boundary between them by means of gravity methods a difficult task^36^. The depth to basement has been estimated by exploiting the global sediments thickness model^37^. In absence of better information a 5 km uncertainty has been considered for this layer.

The Moho depth and its accuracy have been taken from^7^. The last modelled layer is the Curie isotherm, which defines the base of the magnetised lithosphere. The definition of an a-priori Curie depth map is a hard task, since no regional models are available in literature. Here we used the global model from^38^. As for the accuracy we compared the model from^38^ with the Curie depth derived from the WinterC-G model^10^, finding differences up to 25 km. This value has been assumed for the initial Curie depth accuracy. This high uncertainty in the definition of the Curie Point depth is confirmed by comparing the a-priori model with local studies, e.g. in Northern Egypt^39^, Turkey^40^, Adriatic Sea^41^ and Greece^42^. We found in fact differences between^38^ and the local models up to 10 km across the Egyptian coast, 15 km in Turkey, 18 km on the Adriatic Sea and 15 km in Greece. These large discrepancies, are basically due to the fact that Curie Point depth estimation is classically performed by exploiting the relationship between the spectrum of magnetic anomalies and the depth of a magnetic source^43^, without modelling the actual distribution of the magnetic susceptibility.

**Figure 1:**
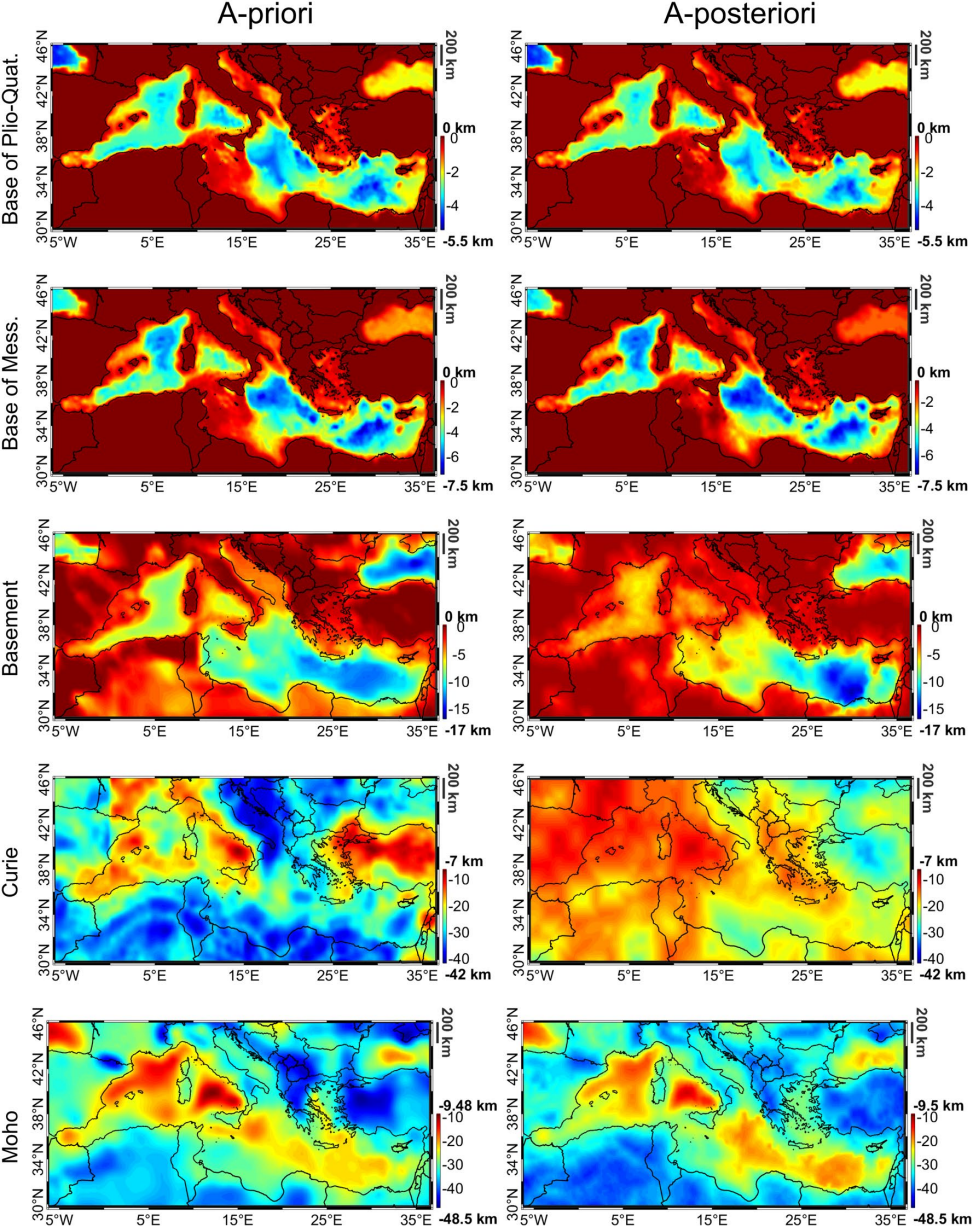
A-priori (left) and estimated geological horizon depth (right). In descending order the horizons are: base of Plio-Quaternary sediments, base of Messinian sediments, depth to basement, Curie and Moho.

The a-priori horizons are reported, for the sake of completeness on Fig. 1, where they are compared with the inversion results.

To complete the a-priori model we need information about the expected density and magnetic susceptibility of each layer. In Tables 1 and 2 the a-priori density and susceptibility models are reported: in particular for each layer we present the mean value (i.e. the expected value at the top of the geological unit), the gradient with depth (supposing a linear dependency) and the expected variability ($\sigma$). As for the densities and susceptibilities variability it describes the range in which the inversion algorithm can change the initial value of each cell of the volume. Since we suppose the density/susceptibility distribution of each volumetric element to be normally distributed with an average given by its mean expected value and a variance given by $\sigma ^2$ we can expect densities/susceptibilities variations in the range $\pm 3 \sigma$ in the inversion results. Note that the Crust layer as parameterised in Table 1 encompasses the magnetised and the non-magnetised crystalline crust - i.e. the geological units 5 and 6.

**Table 1:** A-priori model in terms of density, expressed as average values, linear gradient and variability.

Layer	Mean [kg/m$^3$]	Density gradient [kg/m$^3$/km]	Density variability $\sigma$ [kg/m$^3$]
Water	1030	0	0
Plio-Quaternary	2200	0	22.4
Messinian	2170	0	14
Pre-Messinian	2400	7	26.5
Crust	2890*	3	32
Mantle	3321*	0	32

$^{*}$ The crust and upper mantle lateral density distributions have been taken from^10^ and^44^.

**Table 2:** A-priori model in terms of magnetic susceptibility, expressed as average values, linear gradient and variability.

Layer	Mean [$10^{-6}$ SI]	Susceptibility gradient [$10^{-6}$ SI/km]	Susceptibility variability $\sigma$ [$10^{-6}$ SI]
Water	− 13	0	10
Plio-Quaternary	200	0	63
Messinian	− 30	0	10
Pre-Messinian	900	0	150
Magnetized crust	19709$^*$	0	11662
Mantle	0	0	0

$^{*}$ The crust lateral susceptibility distribution has been taken from^45^.

A density gradient (with depth) has been added for the Pre-messinian sediments to simulate sediment compaction and in the crystalline crust according to^46^. Looking to Table 1 we highlight that the Plio-Quaternary density in our model can range between 2130 kg/m$^3$ and 2270 kg/m$^3$ which is well aligned with the values found e.g. in^47^, where a median value of 2130 kg/m$^3$ for the Quaternary sedimentary sequence and of 2280 kg/m$^3$ for the Pliocene group is found. As for the Messinian layer it is mainly constituted from salt and gypsum^30^ and therefore we supposed a standard density for halite^48^ allowing variations in the range between 2128 kg/m$^3$ and 2212 kg/m$^3$. Considering the Pre-Messinian sediments we used average densities from the CRUST1.0 model^49^ and we supposed a density gradient of 7 kg/m$^3$/km. This means that the expected density varies with depth from 2400 kg/m$^3$ at zero level to about 2520 kg/m$^3$ at a depth of 17 km. As for the density uncertainty of this unit, it has been selected in order to allow densities to move up to the maximum value of the CRUST1.0 sedimentary layer: so we have that the Pre-Messinian sediments density ranges between 2320 kg/m$^3$ and 2600 kg/m$^3$. The crystalline crust density distribution has been computed supposing an average density gradient with depth of 3 kg/m$^3$/km and fitting the average crustal density from^10^. Similarly, the mantle density distribution has been taken from^10^. A plot of the a-priori crustal and mantle density distributions is reported in the supplementary material. High variability has been attributed to the crust and upper mantle density with a $\sigma =32$ kg/m$^3$, allowing variation up to $\pm 100$ kg/m$^3$.

As for the magnetic susceptibility, excluding the evaporites and the water bodies, where susceptibility is well known, local direct information are in general missing and characterised by very high variability, see e.g.^41,50,51^. This is reflected in our a-priori model, where, especially for the crust, we have chosen the very smooth model by^45^ with high uncertainty, which allows the crustal layer susceptibility to range between low susceptibility values (close to 0 SI) typical of continental crust to about 0.06 SI, which is the average used for gabbroid or basalt rocks. We rely mainly on^44^ for the sedimentary layers. A plot of the a-priori crustal magnetic susceptibility is reported in the supplementary material.

Within the current inversion remanent magnetisation has not been modelled. This is however justified by the fact that at high temperatures, i.e. above 400 $^\circ$C, remanent magnetisation contributions are unlikely^52^. Accordingly to the WinterC-G model^10^, the 400 $^\circ$C is found at a depth of 15 km in most of the Mediterranean Sea region, thus reducing the probability of large remanent magnetisation effect on the area. This is also confirmed from the global study in^45^, which shows low values of remanent magnetisation in the Mediterranean Sea region: considering the value reported in^45^ and our a-priori model we expect remanent magnetisation to be smaller than 5% (on average) of the total magnetisation.

Concluding the description of the a-priori model, we just want to underline that the most important layers are the crust and the upper mantle, corresponding to about 85% of the total considered volume, while the sedimentary layers show important features only in well localized regions, such as the Nile Delta where they can reach 30% of the considered depth.

## Results

The results of the joint inversion are a set of three coherent volumes describing the crust in terms of geological units, density and magnetic susceptibility distributions. A representation of the three volumes for the a-priori and a-posteriori models, sliced at latitude of $39^\circ$ N is reported in the supplementary material. The inversion has changed both the geometries of the different geological horizons and the density and magnetic susceptibility, keeping sharp the change in density and susceptibility distributions between the different geological horizons. From the a-posteriori volumes it is possible to extract several derived products such as maps of the main geological horizons as modified by the inversion, and averaged maps of density and susceptibility distributions for each specific layer. After analysing the residuals in terms of gravity and magnetic fields, before and after the inversion, we report and present these derived quantities in order to facilitate the analysis of the results.

### Gravity and magnetic fields residuals

**Figure 2:**
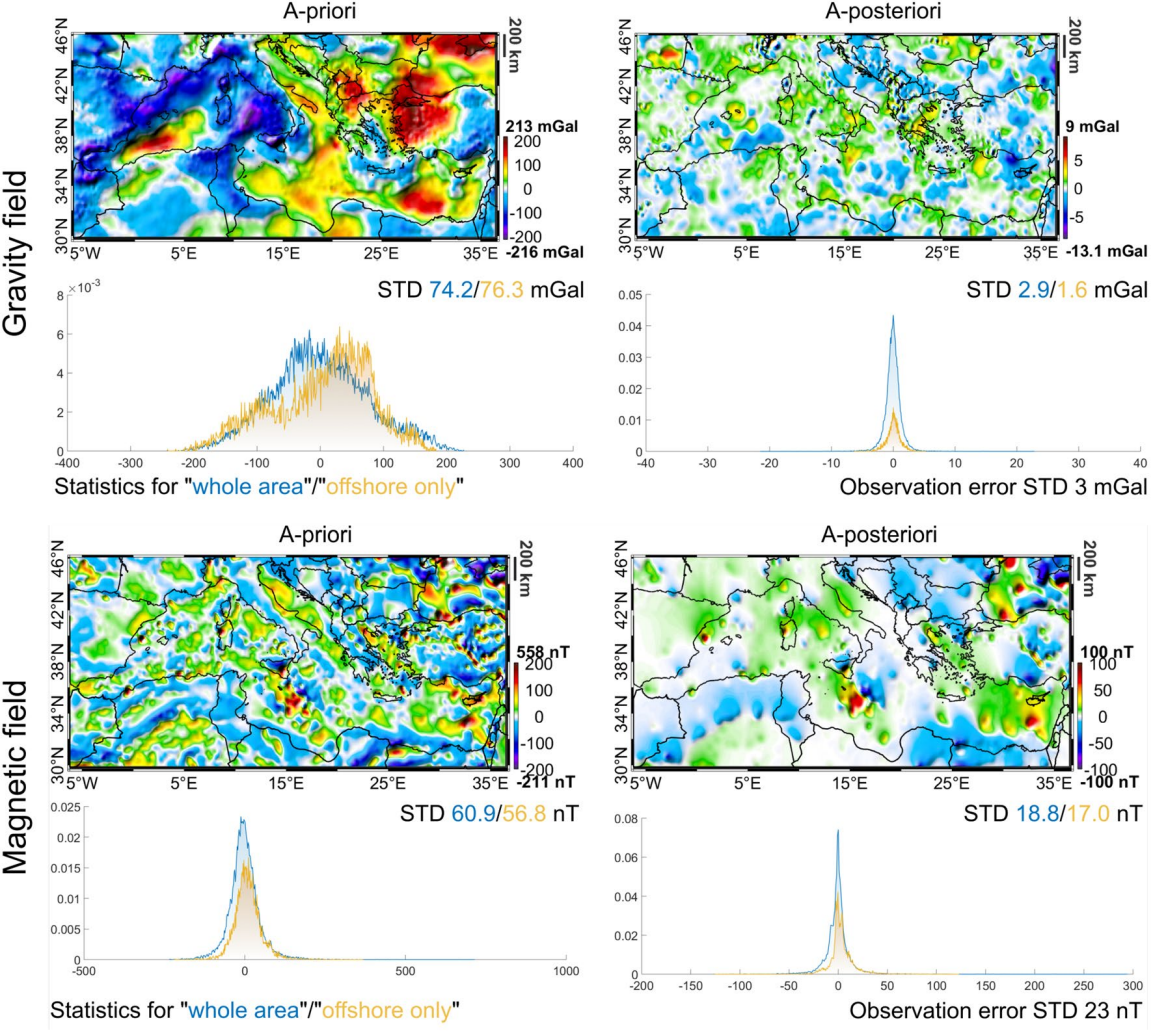
Difference between the observed fields and the effects due to the the model before (a-priori) and after (a-posteriori) the inversion.

Figure 2 shows the difference between the observed gravity field and the gravitational effect of both the a-priori and a-posteriori volumes (i.e. the density distribution before and after the inversion).

We see that the residuals have been notably reduced with just few very localised anomalies close to Cyprus and to the Bay of Biscay where the 99% a-priori confidence interval (9 mGal) is exceeded. So, apart from these few outliers, the inversion explains the observed gravitational signal within the expected a-priori confidence interval. This is confirmed also by the residuals distribution being well symmetric around zero and with an STD of 2.9 mGal decreasing to 1.6 mGal if only the offshore areas are considered. Interestingly, we notice that, for the a-priori model, residuals for the offshore areas are larger than those obtained considering also the onshore, with an STD of 76.3 mGal and 74.2 mGal respectively. This is mainly due to the complexity of the offshore crust combined with lack of good quality constraints in the a-priori Moho (we notice a good correlation between our residuals and the accuracy map in^7^). We observe a general reduction of the residuals for the magnetic field as well, with the STD decreasing from about 60 nT to 18 nT, in agreement with the expected observation error of 23 nT. Relatively high and well localised residuals (e.g. at the boundary between the Levantine and the Herodotus Basins, in correspondence of the boundary of the Ionian Basin and in the Black Sea) remain also after the inversion. These anomalies are located in complex regions at the boundary between different crustal domains where crustal blocks (e.g. the Eratosthenes Sea Mountain Block^53^) generate large magnetic anomalies. The effect of the presence of these blocks, not modelled in our volume, does not cause a distortion of the resulting solution but introduces large discrepancies with respect to the observed field. In order to further improve the fit, it would be necessary to add a-priori information on these blocks and local bodies. This would require a multi-scale approach from large regional to local geology such as the one presented in^54^, which is outside the range of the current study. We also note that these residuals are in relatively cold part of the lithosphere^10^ and therefore remanent magnetisation can play a role too.

What we can derive from this analysis of the residuals is that the obtained a-posteriori model is more coherent with available potential fields observations with respect to the a-priori one, fitting the data with a misfit smaller than the observation errors for both gravity and magnetic fields.

### A-posteriori geological horizons

In Fig. 1 we report the maps of the a-priori and estimated geological horizon depths (while the differences between these two a-posteriori and a-priori maps are reported in the supplementary material). We start our analysis of the retrieved geological horizons by considering the Moho depth.

We notice that the inversion has introduced important variations, up to 8 km, to the a-priori Moho but always in the range defined by the a-priori model accuracy. The main differences are located beneath Turkey and Bulgaria where the algorithm decreased the Moho depth of about 7.7 km, and in Tunisia where a deeper Moho (up to 7.2 km) is found. To validate the solution we performed qualitative and quantitative comparisons with a set of local seismic derived Moho maps^1–5,55–57^. From these comparisons we found that the obtained solution is rich of relevant local-scale features and is in general more similar to the cited local studies than the a-priori one.

**Figure 3:**
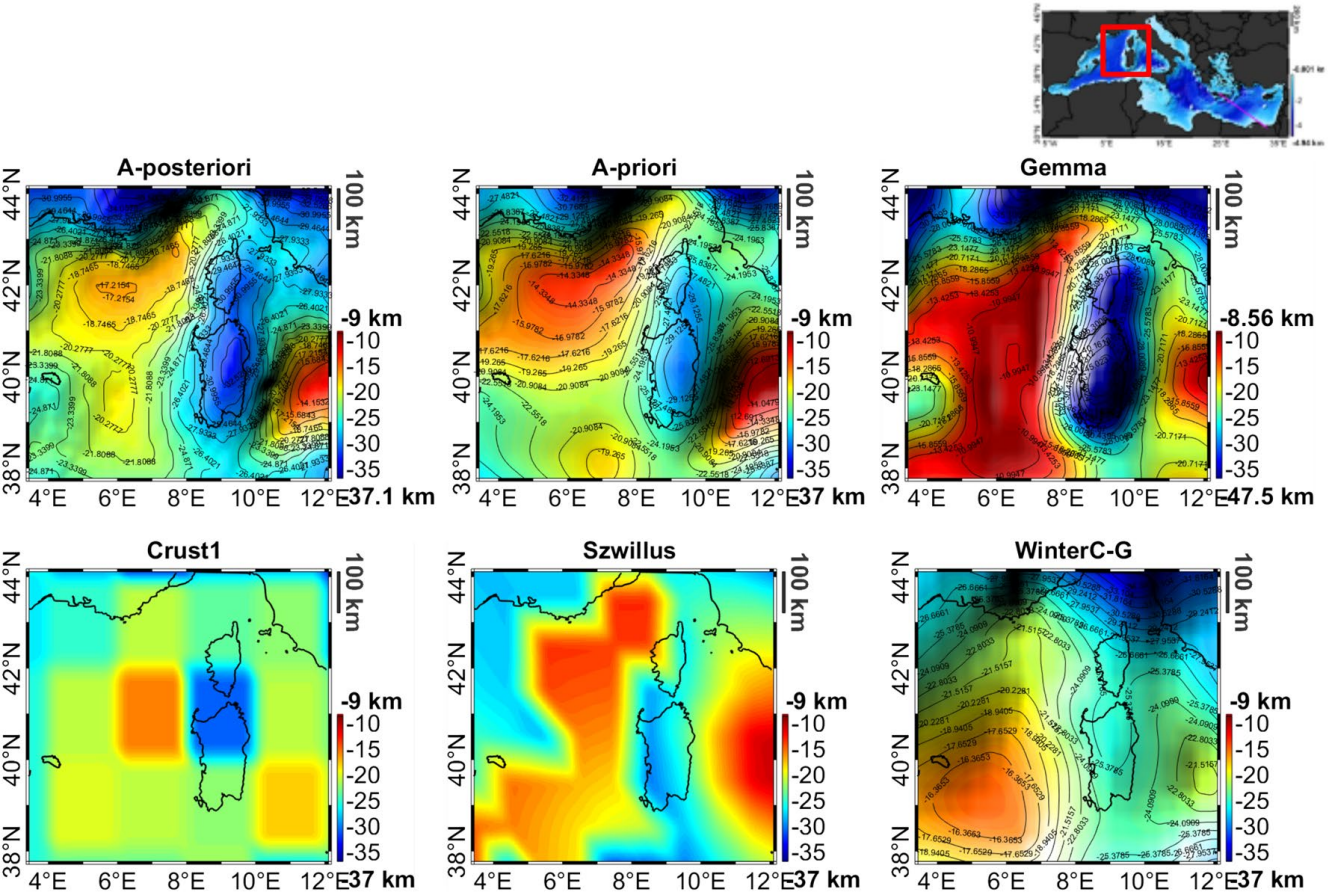
Different Moho maps on the Liguro-Provencal basin.

Focusing for instance on the Liguro-Provencal basin, we qualitatively compared our model with the one published in^2^, which is based offshore on both refraction and reflection data. Since in^2^ Moho depths from ESP seismic data are also indicated for a set of control points, a quantitative evaluation of our solution is also possible. What it turns out is that our results are well aligned with the local Moho model, with depths smaller than 20 km in the oceanic domain, quickly moving to about 30 km below Corsica and Sardinia. Comparing the a-priori and a-posteriori models with the ESP seismic Moho, we obtain a slight reduction of the differences from 2.1 km (STD) of the a-priori model to 1.8 km (STD) of the a-posteriori one. Moreover, if we compare our result with other global Moho models such as CRUST1.0^49^, Gemma^8^, WinterC-G^10^ or the Moho by^9^, the improvement is even more evident (see Fig. 3).

Similar results are found all around the Mediterranean Sea Area, e.g. in the Libyan coast where the STD of differences with respect to seismic derived Moho^55^ drops from 5.2 km of the a-priori model to 3.3 km in the a-posteriori one. Similarly in the Taurus block (Turkey) differences with respects to the seismic profiles in^5^ drops from 5 to 1.6 km (STD) or again in the Tyrrhenian Sea we have that the a-posteriori Moho is more similar to the model found in^57^ than the a-priori one (minimum Moho depth in the area is about 12 km for the a-posteriori and the model by^57^ and about 9 km for the a-priori model).

The estimated depth of Curie isotherm is reported in Fig. 1. We see that the obtained model is in general smoother and shallower than the initial one, with the presence of a shallow (about 10-15 km) Curie in the whole occidental Mediterranean Sea region, reaching 30 km toward the central and oriental Mediterranean Sea basins. We also note a general reduction of the Curie depth in the whole North Africa as well as in the Iberian Peninsula, while a deeper Curie is found in Turkey. This last feature is confirmed by local studies^40^ where a Curie deeper than 20 km in large part of Turkey is found. It is interesting to note that the Mediterranean Sea crust contains pieces of the youngest and oldest oceanic crust worldwide with crust younger than 20 Ma (million years) beneath the Tyrrhenian Sea to crust older than 300 Ma in the Herodotus basin^58^. We therefore confirm the general increase of Curie depths with oceanic crustal ages outlined in^38^: we find a Curie depth of about 10 km in the youngest Tyrrhenian crust, increasing to 15 km in the Liguro-Provencal basin and to more than 20 km in the Herodotus basin.

**Figure 4:**
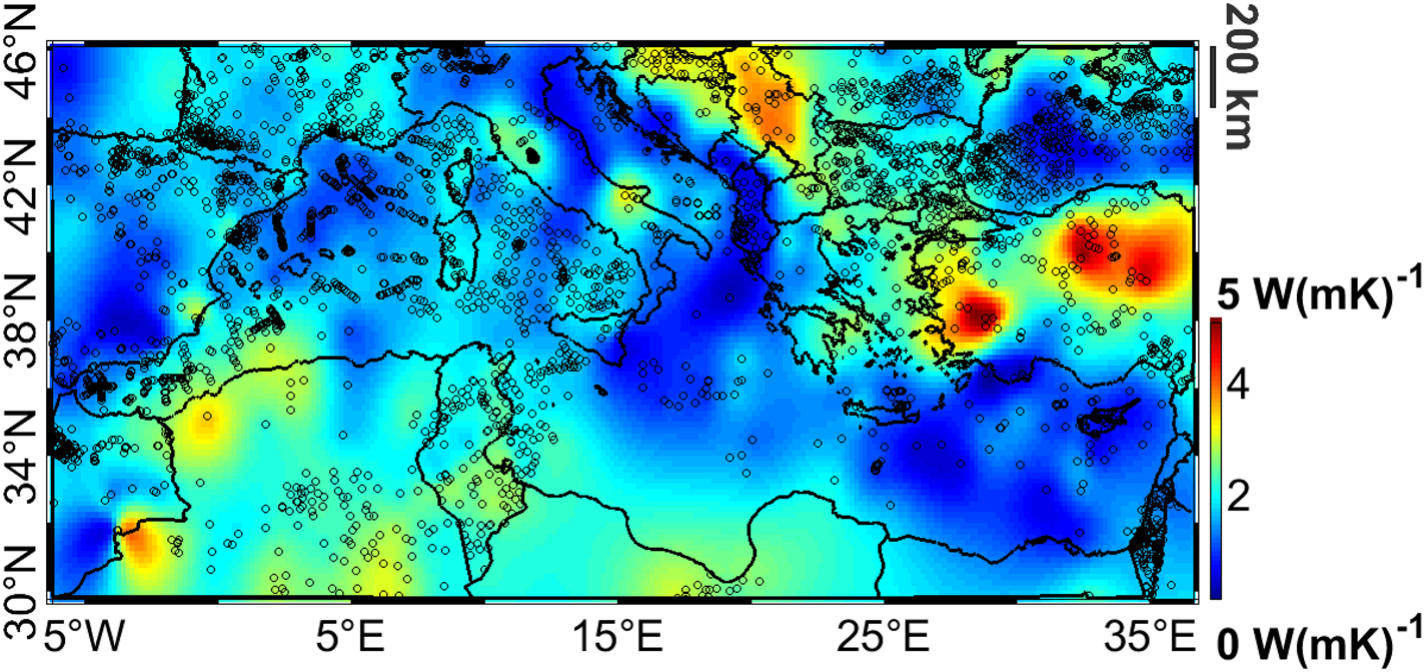
Distribution of thermal conductivity in the Mediterranean Sea area computed accordingly to^38^. Black dots represent the location of heat flow observations.

Since we know that Curie depth and heat flow are correlated^38^, we can exploit the latter to validate the former. We used for the validation the heat flow from^59^, removing outliers, i.e. values larger than 3 times the STD of the dataset itself (about 3$\%$ of the 5140 observations in the study area have been removed) and applying a 25 km moving average to reduce the effect of local anomalies in the data. Comparing the a-priori and the a-posteriori Curie depth maps with the heat flow we obtain a higher correlation of the estimated Curie with respect to the a-priori one (linear correlation coefficients of $-0.55$ and $-0.35$ for the a-posteriori and a-priori models respectively). Starting from the heat flow map and from our Curie depth we can also derive the crustal thermal conductivity (we used the same equations and parameters as in^38^). Correlation between heat flow and Curie depths and theoretical curves with the average thermal conductivity $\lambda$ for different domains are reported in the supplementary material. Figure 4 shows the spatial distribution of the obtained thermal conductivity, black dots are the location of the used heat flow observations.

We find average values of 1.6 W(mK)$^{-1}$ for the young oceanic crust beneath the Tyrrhenian Sea, which are typical values for basalt (see e.g.^60^) and about 1.1 W(mK)$^{-1}$ for the remaining oceanic crust. This last value, which is smaller than typical oceanic crustal conductivity^38^, is mainly affected by the low heat flow of the Eastern Mediterranean Sea. In the continental domain the thermal conductivity is more variable: an average value of 1.6 W(mK)$^{-1}$ is found, which is in the range of the expected values^61^ for continental crust. Note also that the maximum thermal conductivity is found in Turkey, with values similar to those presented in^62^.

Regarding the other layers, we obtain a reduction of the depth to basement offshore up to 4 km, except for some coastal areas close to Crete and Cyprus where an increase of the depth to basement, reaching 2 km, is found (see Fig. 1). Here the most prominent features are the depth to basement lows close to the Nile Delta and beneath the Black Sea (reaching a depth of about 16 km and 12 km respectively). We can also note that the inversion reduced (about 500 m - 1 km) several onshore sedimentary basins, e.g. the Po basin in Italy, the Aquitaine Basin in France, the Ebro, Tajo and Duero in Spain. Apart for the Po basin, the other corrections well correlate with the magnetic residuals of the a-priori model shown in Fig. 2 and therefore they have been probably introduced by the algorithm to fit these data. In any case the obtained depth to basement model (even onshore where the a-priori modelling was not so detailed) fall in the range predicted e.g. by^7^ and is very close to the model presented in^63^. Differences between the a-priori and a-posteriori maps of the base of the Messinian and Plio-Quaternary layers are smaller than 1 km (see Fig. 1) and are partly due to discretization errors (the thickness of our three-dimensional model cells is ranging between 200 m and 600 m at the depth of these two layers). Note that, in any case^18,]^ guarantees that this discretization error does not degrade the inversion results. Main corrections for these two layers are found in the Central and Eastern Mediterranean Sea regions, between Crete and the Levantine where the two layers are known to have important depths.

### Average densities and magnetic susceptibility

**Figure 5:**
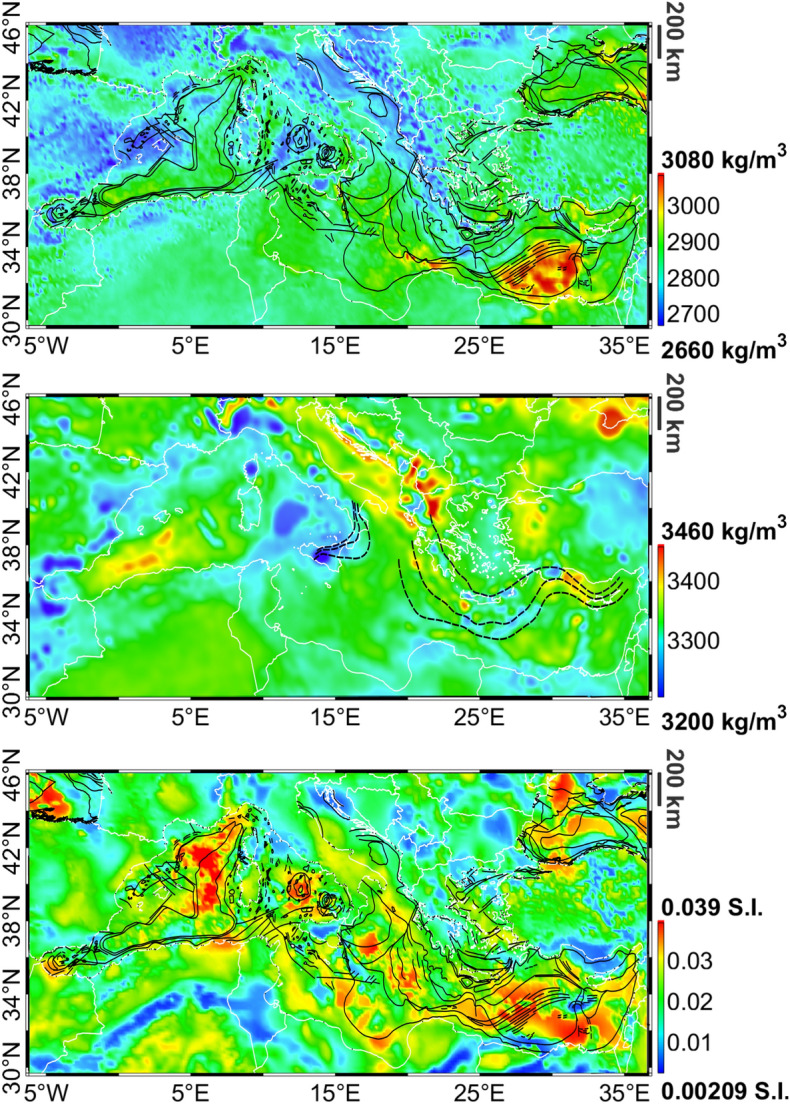
Average estimated crustal (up) and upper mantle (middle) density. Average estimated crustal magnetic susceptibility (down). Black lines represent major faults from^64^, black dashed lines in the middle figure are main subduction geometries between 20 km and 60 km from^65^.

The inversion results are the complete three-dimensional volumes in terms of density distribution and magnetic susceptibility. In order to simplify the visualisation of these results, we computed for each geological unit, the average density and susceptibility in the vertical direction. Note that, thanks to the Bayesian Inversion algorithm adopted in this study, we can easily use the 3D volume in terms of geological units (i.e. the labels) to extract densities and susceptibilities of each layer. Figure 5 shows average density and susceptibility for the magnetised crustal layer.

**Figure 6:**
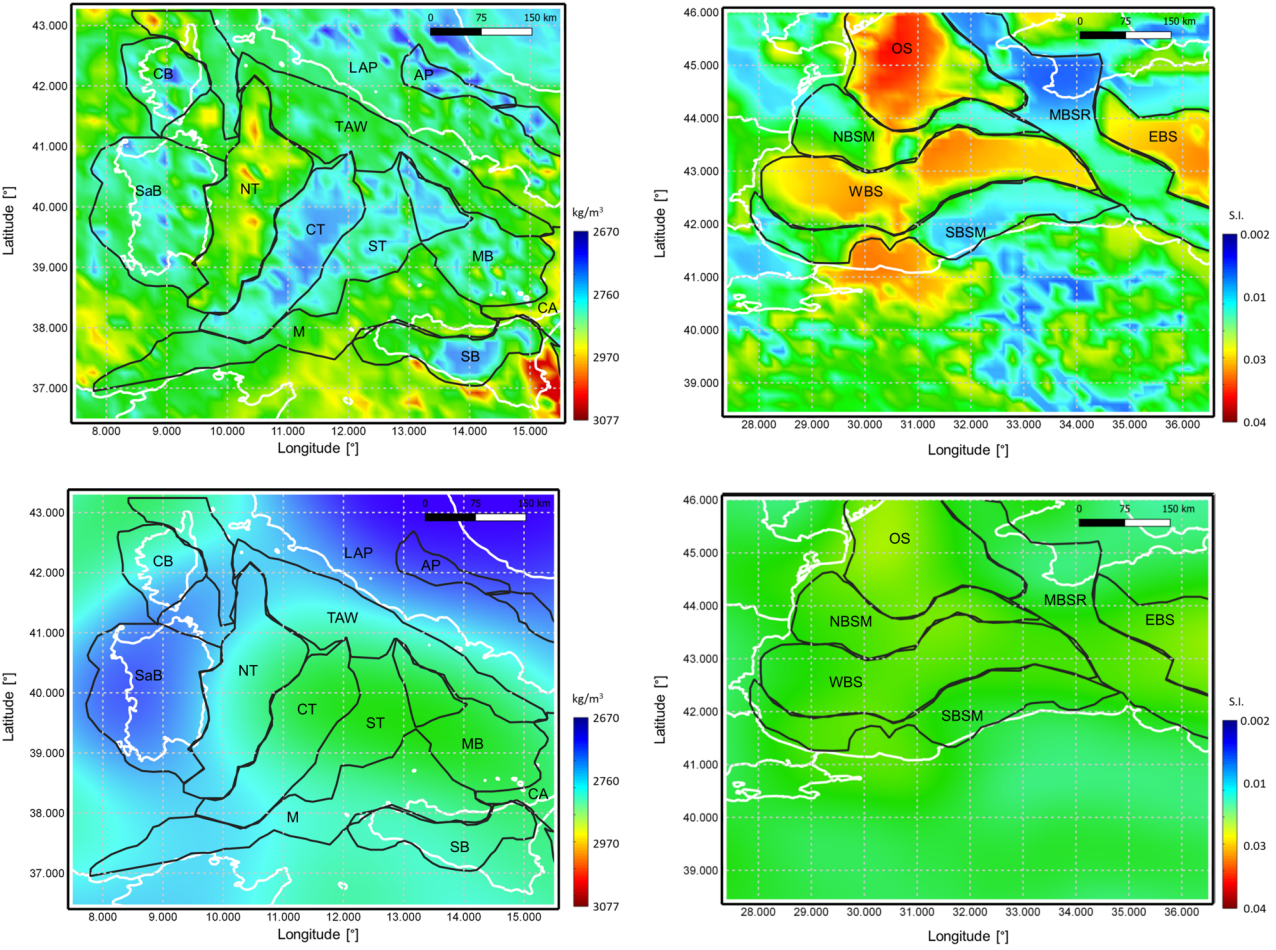
Zoom on the average crustal density map in the Central Mediterranean Sea area (left) and on the average magnetic susceptibility map in the Black Sea area (right). Upper figures report the a-posteriori model, while lower figures are the a-priori one. Black lines outline main basins and crustal domains modified from^64,66,67^, white lines represent coastlines. TAW = Tyrrhenian Accretionary Wedge, SB = Sicily Block, CA = Calabrian Arc, MB = Marsili Basin, ST = Southern Tyrrhenian basin, CT = Central Tyrrhenian basin, NT = Northern Tyrrhenian basin, LAP = Latium Abruzzi Platform, AP = Apennines, M = Magrebides, SaB = Sardinia Block, CB = Corsica Block. WBS = West Black Sea, SBSM = Southern Black Sea Margin, NBSM = Northwern Black Sea Margin, MBSR = Mid Black Sea Rise, EBS = East Black Sea, OS = Odessa Shelf.

We observe that the algorithm has introduced several features: the most evident is the presence of high density oceanic crust in the Herodotus and Ionian basins where the retrieved density reaches values higher than 3000 kg/m$^3$. This corresponds to the oldest oceanic crust (see^58^). High density values (between 2900 and 3000 kg/m$^3$), typical of oceanic crust are found also in the Alborean Sea and in the Liguro-Provencal basins. However for this young oceanic crust, densities are close to those of typical heavy continental crust (such as for instance those found in the Levant Basin) and therefore the distinction on the basis of density alone is difficult. Continental domains are characterised by densities smaller than 2900 kg/m$^3$. We also note that the obtained density distribution correlate well with the main offshore faults from^64^ (black lines in Fig. 5). The obtained solution is rich of relevant local-scale features: focusing as an example on the Central Mediterranean Sea area (Fig. 6, upper-left panel), we identify the Sardinia (SaB) and Corsica (CB) blocks, with densities typical of the European continental crust, a denser crust in the North Tyrrhenian Sea basin (NT), the complexity and fragmentation of the Central and Southern Tyrrhenian basins (CT and ST respectively) and of the Marsili Basin (MB). Boundaries of the Sicily block (SB), of the Calabrian Arc (CA), the Appenines (AP) and Magrebides (M) can be also easily detected and outlined. All these elements were not present in the a-priori model (Fig. 6, lower-left panel), which is characterised just by a smooth density distribution.

Considering the average crustal susceptibility (Fig. 5), we notice high susceptibility values in correspondence with oceanic domains, while continental crust is characterised by a large variability. The map shows the presence of several interesting features such as the distinction between the Northern Tyrrhenian domain and the Central, Southern Tyrrhenian basins, or the southern border of the Atlas Uplift in Algeria appearing as a zero-susceptibility arc in the map. Focusing again on a specific region, such as for instance the Black Sea area (Fig. 6, upper-right panel) several local features are visible, such as the two lobes of oceanic crust of the Western and Eastern Black Sea (WBS and EBS respectively on the map) reaching a susceptibility of about 0.03 S.I., separated by the Mid Black Sea Rise (MBSR - with susceptibility values lower than 0.02 S.I.) and bounded by the Northern and Southern Black Sea Margin (NBSM and SBSM) and the Odessa Shelf (OS), characterised by high values of magnetic susceptibility (larger than 0.03 S.I.) required in order to fit the Odessa Magnetic anomaly. Also for the magnetic susceptibility, the a-priori model contains just an almost constant value of 0.022 S.I.. Looking to the average crustal density of the upper mantle, Fig. 5, we notice the presence of several low-density regions e.g. close to the Strait of Gibraltar to the Tyrrhenian Sea and offshore Crete. These regions, which correspond to low-velocities regions in several seismic tomography models such as^68,69^ are related to main subduction zones (see the geometries of the Calabrian and Hellenic arcs from^65^ reported in the figure).

## Conclusions

We performed a 3D Bayesian inversion of magnetic and gravity anomaly measurements using an extensive database of a-priori information on a regional scale over the Mediterranean Sea crust and upper mantle. The inversion result is a three-dimensional model of this region down to a depth of 50 km with a spatial resolution of 15 km. Comparisons with local studies, seismic derived information, heat flow data not exploited in the inversion are used to successfully assess the results in different regions. The final model is a relevant improvement on the study of the Mediterranean Sea crust and can be used in future as background for further local analysis and studies. The whole model, i.e. the top and the bottom of each layer and the density and magnetic susceptibility distributions is available in the supplementary information.

## Supplementary Information

Supplementary Information 1.

Supplementary Information 2.

## Data Availability

The result of the current study are available in the supplementary information. All the inputs are available from the corresponding author on reasonable request.
