# Author Correction: An enhanced view on the Mediterranean Sea crust from potential fields data

**DOI:** 10.1038/s41598-023-37289-5

**Published:** 2023-06-20

**Authors:** Daniele Sampietro, Martina Capponi, Erwan Thébault, Lydie Gailler

**Affiliations:** 1 Geomatics Research & Development srl, 22074 Lomazzo, CO Italy; 2 Laboratoire Magmas et Volcans, CNRS, IRD, OPGC, Université Clermont Auvergne, 63170 Aubière, France

Correction to: *Scientific Reports* https://doi.org/10.1038/s41598-023-35282-6, published online 23 May 2023

The Acknowledgements section in the original Article was incomplete.

“This work was partially funded by Geomatics Research & Development srl https://www.g-red.eu/ in the framework of the “eXperimental jOint inveRsioN project”. This work was partly funded by CNES in the framework of the project “Exploitation de la mission Swarm”. We thank the WDMAM working group for making the map available at www.wdmam.org.”

now reads:

This work was partially funded by Geomatics Research & Development srl https://www.g-red.eu/ in the framework of the ESA funded “eXperimental jOint inveRsioN project”. This work was partly funded by CNES in the framework of the project “Exploitation de la mission Swarm”. We thank the WDMAM working group for making the map available at www.wdmam.org.

The original Article has been updated.

