# Supplementary material for: An enhanced view on the Mediterranean Sea crust from potential fields data
Source: Sci Rep. 2023 May 23;13:8298. doi: 10.1038/s41598-023-35282-6 (PMC10206088; doi:10.1038/s41598-023-35282-6)
Supplement: Supplementary file 2 — Supplementary Information 2. [file 41598_2023_35282_MOESM2_ESM.pdf]

Supplementary Information to Mediterranean Sea Crust: an Enhanced View from Potential Fields Data

Daniele Sampietro[1,*,+], **Martina Capponi**[1,+], **Erwan Thébault**[2,+], **and Lydie Gailler**[2,+]

[1]Geomatics Research & Development srl, Lomazzo (CO), 22074, Italy
[2]Université Clermont Auvergne, CNRS, IRD, OPGC, Laboratoire Magmas et Volcans, Clermont-Ferrand, France
[*]daniele.sampietro@g-red.eu
[+]these authors contributed equally to this work

Joint gravity and Magnetic Bayesian Inversion

The inversion is based on the methodology described in[1] and updated for the joint gravity and magnetic field in[2]. We report here only the main concepts referring the interested reader to the above references. The most important aspect of the inversion regards the possibility to estimate, at the same time, the 3D density and magnetic susceptibility distributions and the geometrical shapes of main geological units in the given region, respecting all the available constraints. In our method, the studied volume is divided into a set of volumetric elements (voxels): we will suppose to work in planar approximation dividing the volume into right rectangular prisms. This assumption has been checked for the Mediterranean Sea in[3]. For each voxel V_i, with index $i = 1, 2, \ldots, N$, we define three unknown parameters: its density ρ_i, its magnetic susceptibility χ_i and its label L_i. This last variable is used for assigning to V_i a certain geological unit, chosen from an *a-priori* set (e.g., water, sediment, salt, crust, etc.). We will call $\mathbf{x}$ the set of all the unknown parameters, namely, $\mathbf{x} = \{\rho_i, \chi_i, L_i\}\, i = 1 \ldots N$. It is then possible to summarise available regional studies, regional maps, scientific publications, seismic images (2D profiles or 3D volumes), seismic velocities (2D–3D, reflection–refraction), well logs, etc., in terms of an a-priori 3D structural model of x. Even more, in agreement with geological evidences, it is also possible to introduce information on the level of uncertainty about this initial model that can be used to build an a-priori probability function $P(\mathbf{x})$ for the unknown parameters $\mathbf{x}$. Once $P(\mathbf{x})$ is set, we can exploit the Bayes theorem in its classical form:

$$P(\mathbf{x}|\mathbf{y}) \propto \mathscr{L}(\mathbf{y}|\mathbf{x})\, P(\mathbf{x}) \tag{1}$$

to obtain the posterior distribution $P(\mathbf{x}|\mathbf{y})$ of $\mathbf{x}$, given the observations $\mathbf{y}$. In Equation (1), $\mathscr{L}$ represents the likelihood, i.e., the probability of observing $\mathbf{y}$ given $\mathbf{x}$, or, in other words, the compatibility of the evidence with the given hypothesis. The posterior distribution in the inversion of gravity and magnetic fields presented in our work reads:

$$\begin{aligned} P(\boldsymbol{\rho}, \boldsymbol{\chi}, \mathbf{L}|\boldsymbol{\Delta g}^o, \boldsymbol{\Delta B}^o) \propto \exp\Big\{ &-(\boldsymbol{\Delta g}^o - \mathrm{A}_g\boldsymbol{\rho})^T \mathrm{C}_{\Delta g}^{-1} (\boldsymbol{\Delta g}^o - \mathrm{A}_g\boldsymbol{\rho}) - (\boldsymbol{\Delta B}^o - \mathrm{A}_B\boldsymbol{\chi})^T \mathrm{C}_{\Delta B}^{-1} (\boldsymbol{\Delta B}^o - \mathrm{A}_B\boldsymbol{\chi}) - \\ &- \frac{1}{\boldsymbol{\sigma}^2_{\rho_\ell}} (\boldsymbol{\rho} - \overline{\boldsymbol{\rho}}_\ell)^T (\boldsymbol{\rho} - \overline{\boldsymbol{\rho}}_\ell) - \frac{1}{\boldsymbol{\sigma}^2_{\chi_\ell}} (\boldsymbol{\chi} - \overline{\boldsymbol{\chi}}_\ell)^T (\boldsymbol{\chi} - \overline{\boldsymbol{\chi}}_\ell) - s^2(\boldsymbol{L}, \boldsymbol{\ell}^o) - q^2(\boldsymbol{L}) \Big\} \cdot \\ &\cdot \delta_{[\overline{\rho}_\ell | 3\sigma^2_{\rho_\ell}]}(\boldsymbol{\rho})\, \delta_{[\overline{\chi}_\ell | 3\sigma^2_{\chi_\ell}]}(\boldsymbol{\chi}) \end{aligned} \tag{2}$$

where $\boldsymbol{\Delta g}^o$ and $\boldsymbol{\Delta B}^o$ are the vectors of observed gravity and magnetic anomalies with error covariance matrices $\boldsymbol{C}_{\Delta g}$ and $\boldsymbol{C}_{\Delta B}$. A_g and A_B are the forward modelling operator from densities to gravity anomalies and from magnetic susceptibility to total magnetic field intensity. $\boldsymbol{\rho}$ and $\boldsymbol{\chi}$ are the set of densities and magnetic susceptibilities. $\overline{\boldsymbol{\rho}}_\ell$ and $\boldsymbol{\sigma}^2_{\rho_\ell}$ represent the set of expected densities and their variability for the label ℓ and $\overline{\boldsymbol{\chi}}_\ell$ and $\boldsymbol{\sigma}^2_{\chi_\ell}$ are analogous terms for the magnetic susceptibility. Finally s and q are regularising factors acting on the geological unit terms. We used $\boldsymbol{\ell}^o$ to indicate the set of labels in the a-priori model and the subscript ℓ the dependency of the variable from L_i. Equation (2) is highly non-linear, non-convex and contains a huge number of unknowns, so it is particularly difficult to be sampled or even plotted. Due to these factors, the chosen solution is to find the set of parameters $\boldsymbol{\rho}$, $\boldsymbol{\chi}$ and $\boldsymbol{L}$ maximising the posterior distribution.

This will be performed by means of a simulated annealing algorithm (SA) combined with a Gibbs sampler[4]. For a more detailed description of the above optimisation algorithm, the interested reader can refer to Sections 9.6 and 9.7 of[1]. Since the

sampling of $\boldsymbol{\rho}$, $\boldsymbol{\chi}$ and $\boldsymbol{L}$ is a random process, it is possible to repeat the simulated annealing procedure several times starting from different random seeds. This is equivalent to performing a set of independent inversions, each arriving to a slightly different 3D model (in principle) close to the actual absolute MAP. The final result is computed by taking the mode of the label of each voxel, and the average of the density and magnetic susceptibility. Moreover, the frequency of $\boldsymbol{L}$ and the standard deviation of the extracted densities and susceptibilities give a voxel-wise estimate of the accuracy of the retrieved model.

References

1. Sansó, F. & Sampietro, D. Analysis of the gravity field: Direct and inverse problems (2021).

2. Sampietro, D., Capponi, M. & Maurizio, G. 3d bayesian inversion of potential fields: The quebec oka carbonatite complex case study. *Geosciences* **12**, 382 (2022).

3. Sampietro, D., Capponi, M., Thébault, E. & Gailler, L. An empirical method for the optimal setting of the potential fields inverse problem. *Geophys. Prospect.* (2022).

4. Rossi, L. *Bayesian gravity inversion by Monte Carlo methods* (Ph.D. Thesis, Politecnico di Milano, Milano, Italy, 2017).

Representation of the three volumes for the a-priori and a-posteriori models, sliced at latitude of 39◦ N

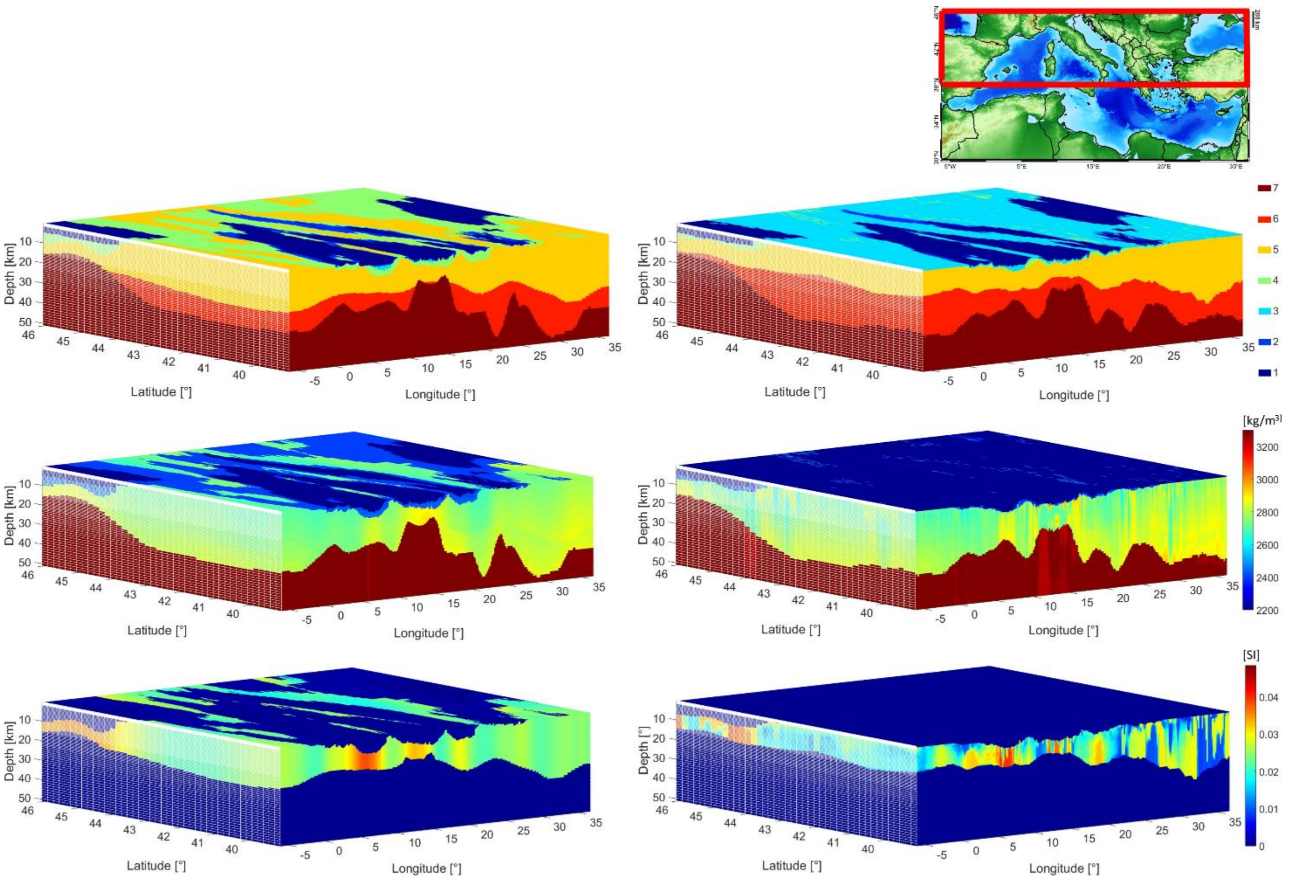

Figure 1. 3D models of the geological unit, density and magnetic susceptibility. The model is shown up to a latitude = 39° North to emphasize the crustal variations beneath the Mediterranean Sea. In the geological units volumes 1 = water, 2 = Plio-Quaternary sediments, 3 = Messinian Sediments, 4 = Pre-Messinian sediments, 5 = magnetized crystalline crust, 6 = non-magnetized crystalline crust, 7 = upper mantle. Left column is the a-priori model, right column is the a-posteriori one.

A-priori density and magnetic susceptibility distributions in the crust and upper mantle

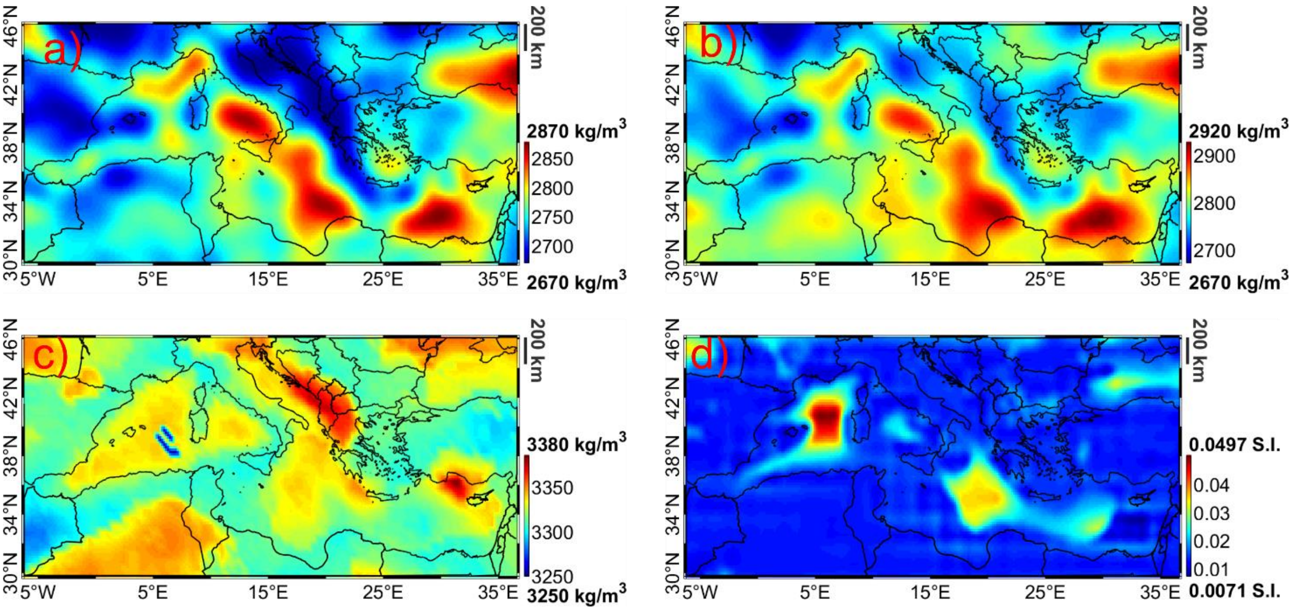

Figure 2. A-priori Density distribution at the top of the magnetized crust a), at the top of non-magnetized crust b), and in the upper mantle c). A-priori magnetic susceptibility distribution in the magnetized crust d).

Differences Between A-posteriori and A-priori geological horizons

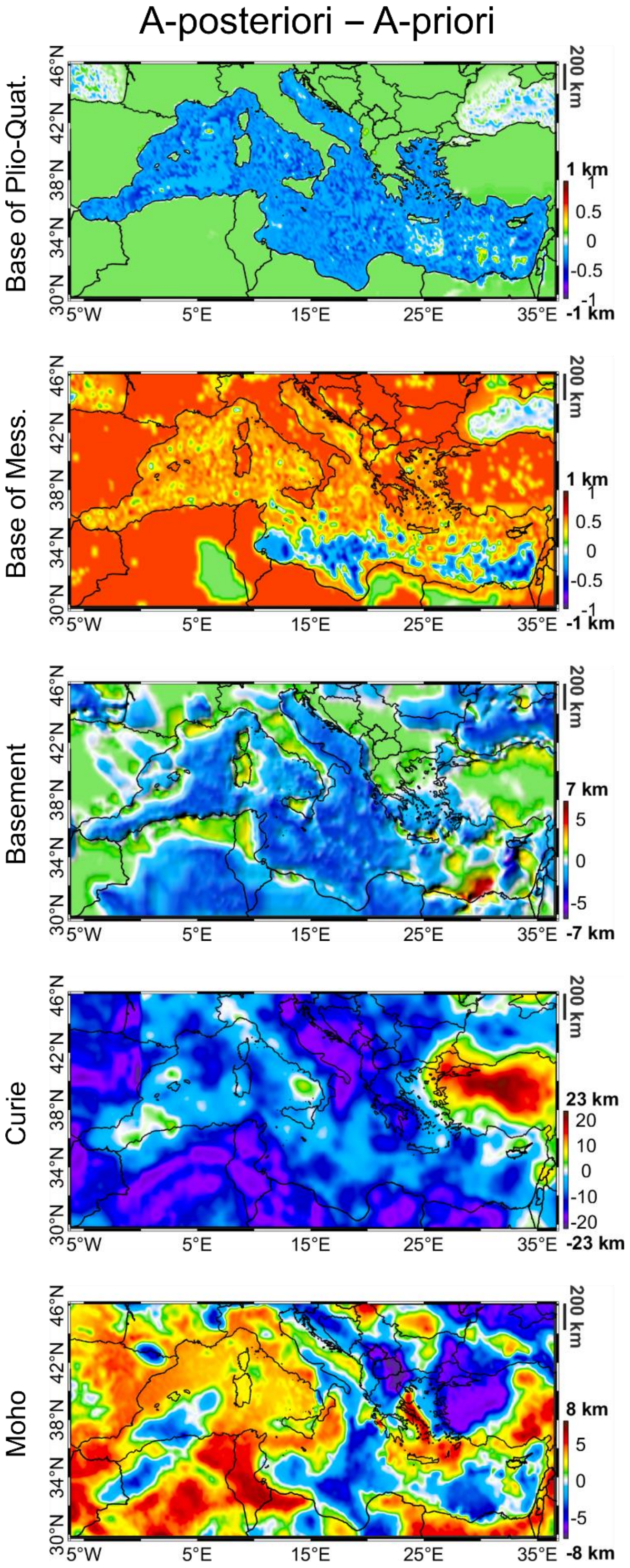

Figure 3. differences between the a-posteriori and a-priori geological horizons depths. In descending order the horizons are: base of Plio-Quaternary sediments, base of Messinian sediments, Basement, Curie and Moho.

Correlation between heat flow and Curie depths and theoretical curves with average thermal conductivity

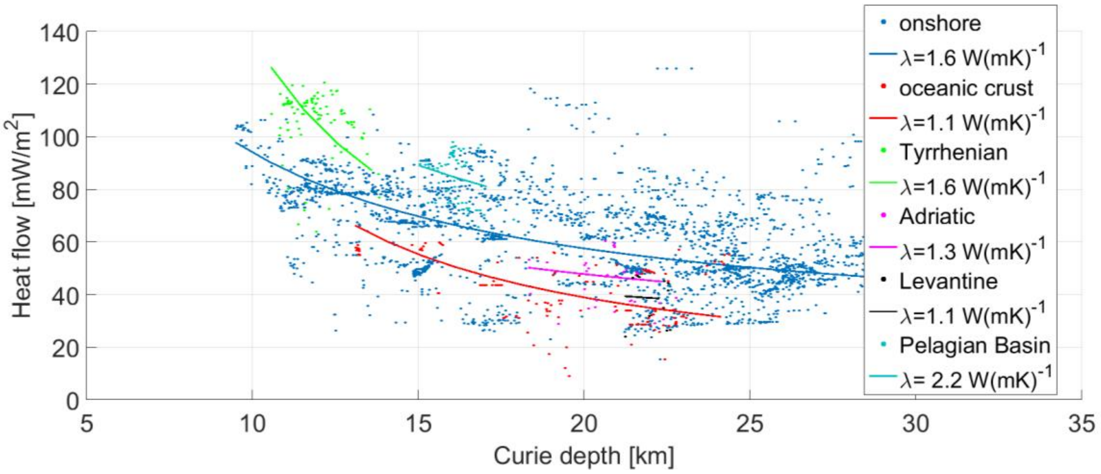

Figure 4: Correlation between heat flow and Curie depths and theoretical curves with average thermal conductivity λ computed accordingly to Li et al. 2017 for different domains.
